# Folate Receptor β (FRβ) Expression on Myeloid Cells and the Impact of Reticuloendothelial System on Folate-Functionalized Nanoparticles’ Biodistribution in Cancer

**DOI:** 10.1021/acs.molpharmaceut.4c00663

**Published:** 2024-08-06

**Authors:** Sibel Goksen, Gamze Varan, Erem Bilensoy, Gunes Esendagli

**Affiliations:** † Department of Medical and Surgical Research, Institute of Health Sciences, Hacettepe University, Ankara 06100, Türkiye; ‡ Department of Vaccine Technology, Hacettepe University Vaccine Institute, Ankara 06100, Türkiye; § Department of Pharmaceutical Technology, Faculty of Pharmacy, Hacettepe University, Ankara 06100, Türkiye; ∥ Department of Vaccinology, Hacettepe University Vaccine Institute, Ankara 06100, Türkiye; ⊥ Department of Basic Oncology, Hacettepe University Cancer Institute, Ankara 06100, Türkiye

**Keywords:** folic acid, cyclodextrin, drug delivery, active targeting, FOLR2, folate receptor

## Abstract

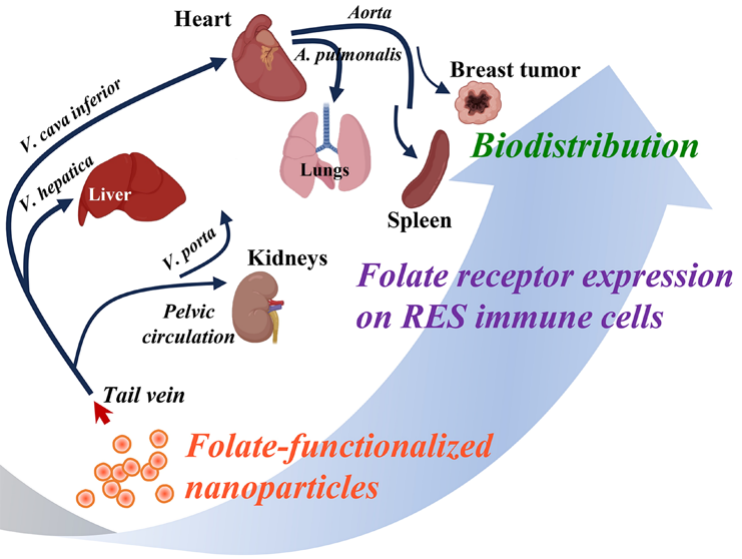

Folate uptake is largely mediated by folate receptor (FR)β, encoded by FOLR2 gene, in myeloid immune cells such as granulocytes, monocytes, and especially in macrophages that constitute the reticuloendothelial system (RES) and infiltrate the tumor microenvironment. Since the myeloid immune compartment dynamically changes during tumorigenesis, it is critical to assess the infiltration status of the tumors by FRβ-expressing myeloid cells to better define the targeting efficacy of folate-functionalized drug delivery systems. On the other hand, clearance by RES is a major limitation for the targeting efficacy of nanoparticles decorated with folate. Therefore, the aims of this study are (i) to determine the amount and subtypes of FRβ^+^ myeloid cells infiltrating the tumors at different stages, (ii) to compare the amount and subtype of FRβ^+^ myeloid cells in distinct organs of tumor-bearing and healthy animals, (iii) to test if the cancer-targeting efficacy and biodistribution of a prototypic folate-functionalized nanoparticle associates with the density of FRβ^+^ myeloid cells. Here, we report that myeloid cell infiltration was enhanced and FRβ was upregulated at distinct stages of tumorigenesis in a mouse breast cancer model. The CD206^+^ subset of macrophages highly expressed FRβ, prominently both in tumor-bearing and healthy mice. In tumor-bearing mice, the amount of all myeloid cells, but particularly granulocytes, was remarkably increased in the tumor, liver, lungs, spleen, kidneys, lymph nodes, peritoneal cavity, bone marrow, heart, and brain. Compared with macrophages, the level of FRβ was moderate in granulocytes and monocytes. The density of FRβ^+^ immune cells in the tumor microenvironment was not directly associated with the tumor-targeting efficacy of the folate-functionalized cyclodextrin nanoparticles. The lung was determined as a preferential site of accumulation for folate-functionalized nanoparticles, wherein FRβ^+^CD206^+^ macrophages significantly engulfed cyclodextrin nanoparticles. In conclusion, our results demonstrate that the tumor formation augments the FR levels and alters the infiltration and distribution of myeloid immune cells in all organs which should be considered as a major factor influencing the targeting efficacy of nanoparticles for drug delivery.

## Introduction

In cancer, the cells of the immune system become phenotypically and functionally altered not only in the tumor microenvironment but also systemically in distinct organs.^1,2^ Especially, the myeloid cells, which are also major components of the reticuloendothelial system (RES), tend to infiltrate various organs and favor tumor progression.^3,4^ Elevated numbers of myeloid cells such as granulocytes, monocytes, and macrophages have been acknowledged as a common facet of cancer.^5^ Nevertheless, myeloid subpopulations display heterogeneity, have immunosuppressive capacities, promote angiogenesis, invasion, and metastasis.^6,7^ The metabolic pathways are also drastically modulated under the influence of cancer.^8^ Folate (vitamin B9) metabolism, which is critical for nucleotide synthesis, mitochondrial ribonucleic acid (RNA) modification, amino acid homeostasis, and methylation of biomolecules, is essential for cell proliferation, mitochondrial energy pathways, and epigenetic regulation.^9^ Not only cancer cells but also immune cells infiltrating the tumor tissue have higher folate requirement for deoxyribonucleic acid (DNA) synthesis, transcription machinery, and repair mechanisms.^10^ Therefore, folate uptake mechanisms by the immune cells are upregulated in cancer.^11−13^

The folate receptor (FR) family consists of 4 isoforms, including FRα, FRβ, FRγ, and FRδ.^14,15^ These receptors differ in tissue/cell-specific distribution and folate binding capacity.^16^ FRα is specifically expressed in tumor cells of epithelial origin,^17−19^ whereas FRβ is frequently expressed in macrophages. FRδ is specifically expressed by oocytes and regulatory T (Treg) cells.^14,15^ Upon binding to folate, FRs are rapidly internalized, and release folate into the cytoplasmic compartment and then recycled to cell surface.^20−22^

FRβ, which is encoded by FOLR2 gene, binds folate with the highest affinity (*K*_d_ ∼ 0.1–1 nM) when compared to other transporters for folate.^23^ It is expressed on activated macrophages, tissue-resident macrophages, and tumor-associated macrophages (TAMs).^23−25^ Additionally, M2 subtype of macrophages has greater interest to folate than M1 subtype.^25^ During acute infectious diseases, macrophages polarize into classically activated M1 subtype.^26^ M1 macrophage-mediated inflammatory responses are distinguished with enhanced microbicidal capacity and increased production of pro-inflammatory cytokines and radicals.^27^ Macrophage-mediated antitumor immunity has been attributed to the M1 subtype.^28^ In contrast, M2 macrophages, which are also known as alternatively activated macrophages, resolve inflammation, contribute to tissue healing, and immune tolerance.^29^ Therefore, FRβ has been regarded as a useful surface molecule not only for phenotyping but also for therapeutically targeting the macrophages or the tissues infiltrated by the macrophages.^12,24^ Especially, depletion or subversion of TAMs, which favor tumor progression and metastasis, into M1-like activated macrophages are promising antitumor approaches.^28^

Many drug delivery strategies have been tested for active targeting of tumors through folate-functionalized nanoparticles.^30,31^ Antitumor efficacy of targeted nanoparticles depends on the physicochemical characteristics of the drug-nanoparticle complex and the biological properties of the target molecule and aimed tissue.^32^ The rapid clearance of nanoparticles by the cells of RES is a major limitation in drug delivery.^33^ Since FRβ is highly expressed by the macrophages and the myeloid cells are usual components of the RES, this study aims to determine the amount of FRβ-expressing myeloid cells (granulocytes, monocytes, and macrophages) and to interrelate the RES escape/circulation time, biodistribution, and tumor-targeting efficacy of folate-decorated cyclodextrin nanoparticles in a mouse breast cancer model, in vivo. This study reports two main findings: (i) as the tumor grows, it becomes progressively infiltrated by the myeloid cells that express FRβ that converts the tumor tissue into a preferential site for active targeting with folate-functionalized nanoparticles. Conversely, (ii) the amount of FRβ^+^ myeloid cells is also increased almost in all organs of the tumor-bearing animals and hampers the delivery of nanoparticles into the tumor.

## Materials and Methods

### Animals and Tumor Model

4T1 breast tumor has been regarded as a suitable experimental animal model for human breast cancer. It is a very aggressive type of cancer which significantly affects the immune system including the myeloid compartment.^34^ The breast cancer model was established in BALB/c mice (6–8 weeks, 18–22 gr) (Kobay AS). The 4T1 breast cancer cell line (American Type Culture Collection, LGC Promochem) was cultured in RPMI 1640 medium (Biowest) supplemented with 1% penicillin/streptomycin (Biological Industries), and 10% fetal bovine serum (FBS; Biological Industries) in humidified atmosphere with 5% CO_2_ at 37 °C. 4T1 cells (5 × 10^4^) were subcutaneously inoculated into the left-inguinal mammary fat pad of BALB/c mice. The animals were maintained under a constant temperature (23 ± 2 °C), humidity (50%), and filtered air. Tumor size and animal weight were measured biweekly. Geometric mean of the tumor width and length was calculated. All of the experiments and handling of animals were performed following approval by the local ethics committee before the commencement of the animal experiments (approval no.: 2017/59-06).

### Reverse-Transcriptase Polymerase Chain Reaction (RT-PCR)

RNA was isolated from 4T1 cell line and 4T1 tumors with a Nucleospin RNA kit (Macherey-Nagel) and cDNA synthesis was performed (ProtoScript II kit, NEB). RT-PCR was performed with the primer oligonucleotide sequences for house-keeping B-actin gene (Actb, NM_007393.5), forward primer 5′-CACTGTCGAGTCGCGTCC-3′, reverse primer 5′-TCATCCATGGCGAACTGGTG-3′; for Folr1 gene (NM_001252552.1), forward primer 5′-TGGAGTTGGCGATTAGAGGTC-3′, reverse primer 5′-CAGGGCCCGGTTTTTCTTTG-3′; for Folr2 gene (NM_001303239.1), forward primer 5′-GTGGACCAGAGTTGGCGTAA-3′, reverse primer 5′-GGGCACTTGTTAATGCCTGAG-3′; for Folr4 gene (NM_022888.2), forward primer 5′-ACGAACTCTACCAGGAGTGCAG-3′, reverse primer 5′-GTTGGGGGAACACTCATGGA-3′. PCR products were documented under UV light (ChemiDoc Imaging Systems, Biorad) after agarose gel electrophoresis and ethidium bromide staining.

### Cell Isolation

Following scarification of the animals, tumor, lungs, liver, spleen, brain, heart, kidneys, and inguinal lymph nodes were removed and placed in RPMI 1640 medium. Bone marrow was flushed from the femur and tibia bones with 0.9% saline solution, and peritoneal lavage was performed with injection of cold phosphate-buffered saline (PBS). The organs were minced mechanically and taken into enzymatic digestion solution containing collagenase type II (100 U/mL, Nordmark) and DNase I (200 U/mL, Sigma-Aldich) in RPMI 1640 were and agitated at 37 °C until dissociation (approximately for 1 h). Then, the dissociated tissue was passed through 40 μm pore-sized filters (SPL Life Sciences) to obtain a single cell suspension.

### Flow Cytometry

The cells were labeled with antimouse CD45 (30-F11), CD11b (M1/70), F4/80 (BM8), CD206 (C068C2), Gr-1 (RB6-8C5), and FRβ (10/FR2) fluorescently labeled monoclonal antibodies which were purchased from BioLegend or Sony Biotechnology. Following the incubation with specified antibodies, the cells were washed and analyses were performed on a flow cytometer (FACSCanto II, BD). The gating strategy used is shown in Supporting Figure 1. Briefly, CD45^+^CD11b^hi^ myeloid immune cells were gated from the single events and the cell populations with appropriate cell sizes and granularity. Then, macrophages were detected as the F4/80^+^CD206^–^ and F4/80^+^CD206^+^ populations. Granulocytes and monocytes were depicted as Gr-1^hi^ and Gr-1^lo/mo^ cells, respectively. The percentage and median fluorescence intensity (MFI) values were defined according to the autofluorescence controls. A heat map output was depicted for the MFI or percentage data where appropriate.

### Calculation of FRβ Density in Tissues

The *z*-score formula, *z* = (*x* – μ)/σ, enables comparison of FRβ MFI values of different myeloid cell types across various scenarios. In tumor tissue development, the formula was employed to assess the mean FRβ MFI values of four distinct cell types at different time points. This analysis helps gauge how each cell type’s mean FRβ MFI values (μ) deviate from the overall average (*x*), considering the standard deviation (σ) of the mean FRβ MFI values specific to the respective time points. Moreover, the *z*-score formula was extended to compare the mean FRβ MFI values of myeloid cells infiltrated into different tissues of both healthy and advanced tumor burden animals. In this case, denoted as μ (mean), it deviates from the global mean, denoted as *x*. This evaluation incorporates the standard deviation (σ) of the cell-specific mean FRβ values associated with the infiltration into diverse tissues. In essence, it helps to demonstrate how the average FRβ levels of each cell type differ from the overall average of cells infiltrated into different tissues of healthy and tumor-bearing animals. FRβ expression density for each cell type was calculated with the formula [# cells/mg × FRβ_MFI_ × FRβ^+^ %] for each organ or tissue studied. Then, the average of the “FRβ expression density”, which was specifically calculated for granulocytes, monocytes, macrophages, and CD206^+^ macrophages, was taken and used as a FRβ expression density score.

### Immunofluorescence

Frozen sections of the tumors (5 μm) were blocked (Super Block, ScyTek Laboratories) for 30 min and incubated with antimouse-CD206 (MR5D3, 1/100; Invitrogen) and -Folr2 (PA5-103843; 1/200; Invitrogen) primary antibodies. Antibody binding was visualized by Alexa488- and Alexa555-conjugated secondary antibodies (1:1000, Abcam), following counterstaining with 4′,6-diamidino-2-phenylindole (DAPI, 300 nM; Sigma-Aldrich). The specimens were analyzed under a fluorescence microscope (Olympus) and the images were processed with ImageJ software (NIH Image).

### Preparation and Characterization of Cyclodextrin (CD) Nanoparticles

The CD nanoparticle formulations used in this study were previously synthesized and characterized by our group and detailed information on their properties was previously published.^35−37^ CD derivatives were used to prepare nanoparticles.^36,37^ βCD6 was achieved through the grafting of 12 C chains onto the secondary face of the β-CD glucose units through ester bonds.^38^ A folate-conjugated derivative on the primary face grafted to aliphatic chains was synthesized as a folate-functionalized derivative CD (Ff-CD).^36,37^ The nanoparticles were prepared with smooth spherical surfaces that can maintain their physical stability in terms of mean diameter and ζ-potential for 30 days in an aqueous dispersion state.^35−37^ The CD nanoparticles were prepared by nanoprecipitation and loaded with Nile red as a fluorescence tracker. Briefly, Nile red (20% of CD) was dissolved in ethanol while magnetic stirring at 660 rpm for 30 min. The organic phase (1 mL) was added to the aqueous phase (2 mL). Subsequently, the organic solvent was evaporated under vacuum.^36^ Particle size (nm), polydispersity index, ζ-potential (mV), and the amount of Nile red encapsulated in nanoparticles are shown in Table 1. Particle sizes were identified (Nanosight NS300, Malvern Analytical). Polydispersity index and ζ-potential were determined (Zetasizer NanoSeries ZS, Malvern Instruments) at a 90 °C angle and 25 °C. The quantification of Nile red encapsulated in the nanoparticles was performed through an indirect method. Specifically, the nanoparticles aqueous dispersion underwent centrifugation at 3500 rpm for 15 min to eliminate unloaded free dye, and the resulting supernatant was quantified using UV spectrophotometry (λem: 590 nm, λex: 546 nm). The following equation was used to calculate the associated dye (%) = [experimental dye loading (μg)/theoretical dye loading (μg)] × 100.

**Table 1:** Physicochemical Properties of Cyclodextrin Nanoparticles

	particle size (nm)	polydispersity index (PDI)	ζ-potential (mV)	Nile red encapsulation (mg/mL)
βCD6	108.9 ± 2.9	0.419 ± 0.019	–8.9 ± 0.4	0.03
Ff-CD	106.3 ± 4.1	0.145 ± 0.012	–11.1 ± 0.5	0.036

### In Vivo Imaging

Nile red-loaded cyclodextrin nanoparticles (3 μg of Nile red/mouse) were administered intravenously through the tail vein when the tumor size reached ∼0.5 cm. Following 24 h, the mice were sacrificed under anesthesia (*n* = 3), and the organs (lungs, liver, heart, kidneys, spleen, and tumor) were dissected and put under an in vivo imaging system (Newton 7.0, Vilber). Fluorescence intensity of Nile red was acquired at 580 nm (λem: 636 nm, λex: 552; Nile red) and processed with ImageJ software (Fiji). Subsequently, the organs were dissociated, and the presence of Nile red-loaded nanoparticles were assessed by flow cytometry at the cellular level.

### Statistical Analysis

One-way ANOVA was used for multiple comparisons using GraphPad Prism 8 (GraphPad Software Inc., San Diego, CA). Paired or unpaired two-tailed Student’s t-test was performed where appropriate for assessment of statistical significance. A value of *p* < 0.05 was considered statistically significant. Results were expressed as mean ± SEM (standard error mean).

## Results

### Tumor Progression Augments Myeloid Infiltration and FRβ Expression in the Tumor Microenvironment

**Figure 1:**
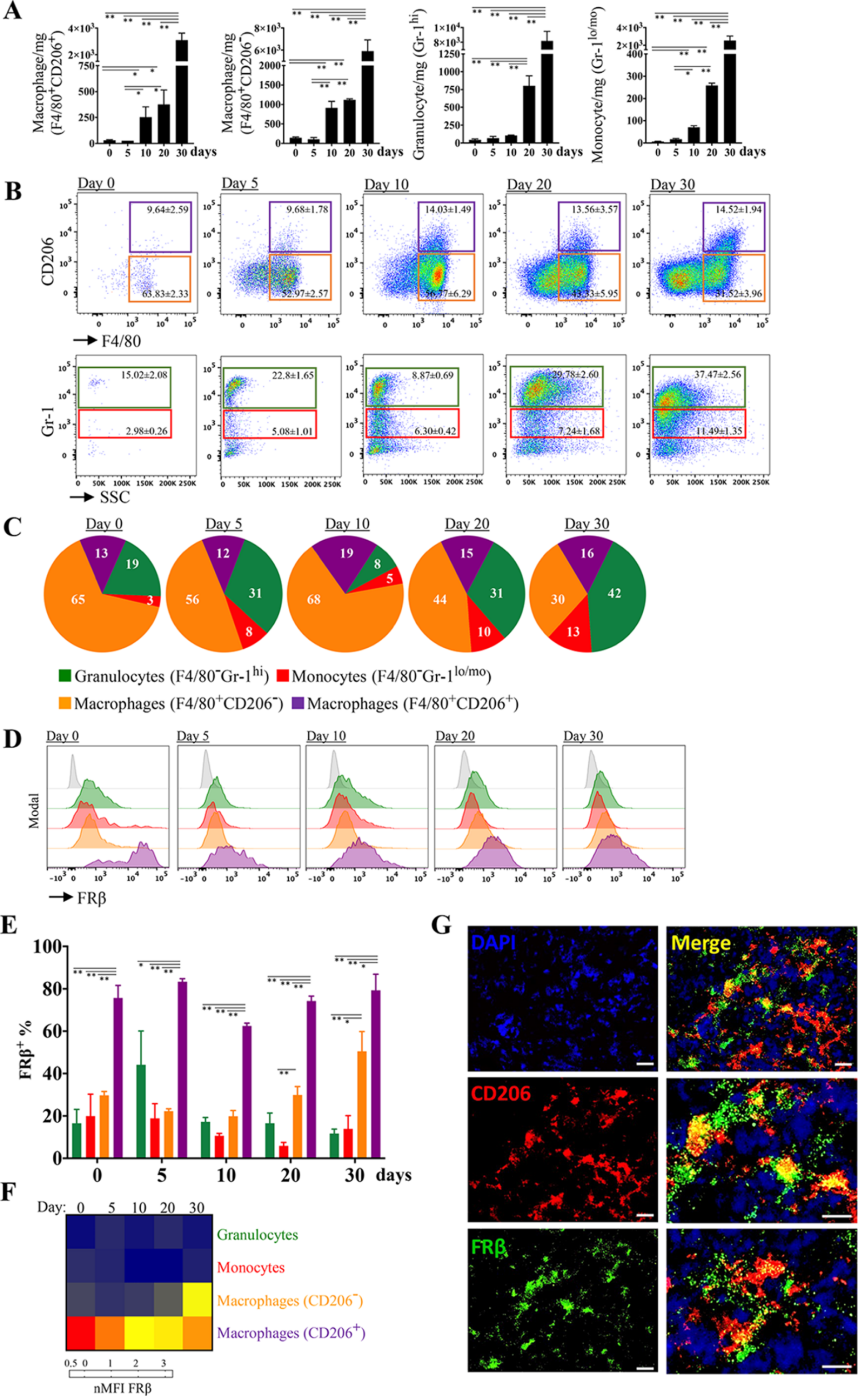
Distribution of myeloid cell subsets and FRβ expression during tumor formation in mammary tissue. The mice were inoculated with 4T1 mammary cancer cells and sacrificed at distinct on days 5, 10, 20, and 30 to obtain whole tumor tissues at distinct phases of growth. The growth curve of 4T1 tumors is supplied in Supporting Figure 2. Day 0 represents the mammary tissue of healthy mice. The tissues were processed, and immunophenotyping analyses of myeloid cells were performed by multicolor flow cytometry. The gating strategy used for flow cytometry is given in Supporting Figure 1. (A) Absolute numbers of CD206^+^ macrophages, CD206^–^ macrophages, monocytes, and granulocytes infiltrating the mammary tissue were analyzed according to the total cell number per mg of tissue and percentages determined by flow cytometric immunophenotyping performed on specific time points following the inoculation of 4T1 cancer cells. (B) Representative flow cytometric scatter plots and percentages of myeloid cells determined under the CD11b^+^ gate. (C) Percentage distribution pie charts showing a dynamic change in the infiltration of myeloid cell subsets at specific time points during 30-day-long tumorigenesis. (D) Representative offset flow cytometry histograms, (E) percentage bar graphs, and (F) median fluorescence intensity (MFI) heat map for FRβ expression on CD206^+^ macrophages, CD206^–^ macrophages, monocytes, and granulocytes on day 0 (healthy mammary tissue), day 5, day 10, day 20, and day 30 of tumorigenesis. (G) The frequent presence of FRβ on CD206^+^ macrophages were validated by immunofluorescence staining of the tumor tissue sections. The middle and lower panels on the right-hand side provide higher magnification of the micrographs (scale bars, 10 μm). The data are presented as average ± SEM. Statistical difference was calculated with Student’s *t* test, (*n* ≥ 4; *p ≤ 0.05, **p ≤ 0.01).

Cancer affects immune functions and hematopoiesis; the cells of myeloid origin increase in number, show immature characteristics, and acquire pro-tumor functions.^29^ In this study, mice bearing 4T1 mammary tumors were used as a typical cancer model wherein myeloid subset of the immune cells is considerably altered.^39,40^ We first determined the time-dependent increase in classical macrophages, CD206^+^ macrophages, granulocytes, and monocytes as cancer progressed. Changes in animals’ weight and tumor size during the follow-up are shown in Supporting Figure 2. The number of myeloid cells, which were obtained in small quantities in mammary fat pads of healthy mice (day 0), began to increase on the fifth day following tumor cell inoculation (Figure 1A). On the fifth day, the percentage of granulocytes among other myeloid cell types was increased at the inoculation site (Figure 1B,C). On day 10, when the tumor reached a palpable size, macrophage dominance (F4/80^+^ total macrophages; range, 46–87% of all myeloid cells) was evidenced (Figure 1C). As the tumor grew, the number of myeloid cells, especially neutrophils, increased (Figure 1A). On the 20th and 30th days of tumorigenesis, granulocytes [804 ± 140 cells/mg (31% of total myeloid cells) and 8184 ± 1272 cells/mg (42% of total myeloid cells), respectively] and monocytes [259 ± 11 cells/mg (10% of total myeloid cells) and 2523 ± 504 cells/mg (13% of total myeloid cells), respectively] were frequently observed in the tumor microenvironment (Figure 1A–C). Interestingly, the proportion of CD206^+^ macrophages remained almost constant (range, 12–19% of total myeloid cells) at all time points tested despite the increase in the number of infiltrating myeloid cells (Figure 1C).

Next, we examined the gene expression of mouse FR isoforms (Folr1 for FRα, Folr2 for FRβ, and Izumo1r for FRδ) in the 4T1 cell line and in the tumors established with 4T1 cells (Supporting Figure 3). Folr1 was slightly expressed in 4T1 cells or in the established tumors. Izumo1r was barely detected in the tumor tissue but not in the cultured 4T1 cell line. On the other hand, Folr2 was highly and exclusively expressed in the tumors but not in the cultured 4T1 cell line which indicated the presence of FRβ in the cells infiltrating the tumor microenvironment (Supporting Figure 3). FRβ expression was particularly prominent in tissue-resident CD206^+^ macrophages in healthy mammary tissue (day 0, 76 ± 6% and 6302 ± 3841 MFI) (Figure 1D–F). With tumorigenesis, CD206^+^ macrophages maintained high levels of FRβ expression (ranges of 63–79% and 1320–6302 MFI). After 20 days, the tumor-infiltrating CD206-negative macrophages also upregulated FRβ (30 ± 4%). Only minor percentages of granulocytes and monocytes expressed FRβ at low levels (Figure 1D–F). The frequent presence of FRβ expression on CD206 macrophages was also verified on the tumor sections (Figure 1G).

Collectively, the myeloid infiltration was significantly increased at the site of tumorigenesis as the tumor progressed; nevertheless, the proportion of CD206^+^ macrophages tended to remain constant. The CD206^+^ macrophage population highly expressed FRβ. At the late stages of tumor formation, FRβ was upregulated by either CD206^+^ or CD206^–^ classical macrophages in the tumor microenvironment.

### Systemic Impact of Cancer on Myeloid Infiltration and FRβ Levels in Various Organs of RES

**Figure 2:**
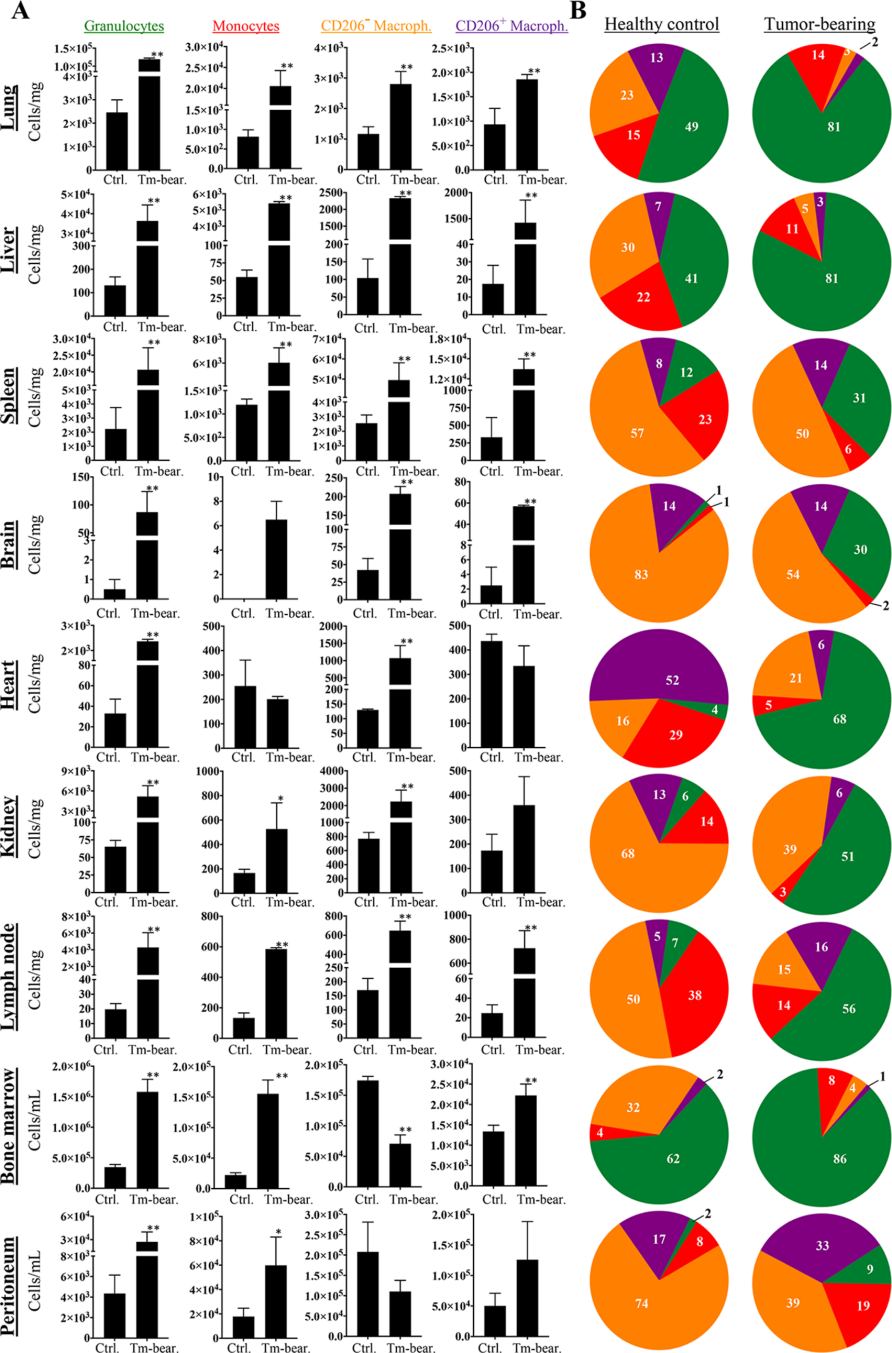
Change in myeloid cell infiltration of distinct organs and compartments in tumor-bearing mice. The cell suspensions were prepared from the tissues collected, and immunophenotyping analyses of myeloid cells were performed by multicolor flow cytometry. The gating strategy used for flow cytometry is given in Supporting Figure 1. (A) Bar graphs show the number of granulocytes, monocytes, CD206^–^ macrophages, and CD206^+^ macrophages in tumor-bearing mice on day 30 and in control healthy mice. (B) Percentage distribution of myeloid cell subsets in the organs of healthy and tumor-bearing mice. The data are presented as average ± SEM. Statistical difference was calculated with Student’s *t* test, (*n* = 6; *p ≤ 0.05, **p ≤ 0.01).

Another aim of this study was to compare the amount and subtype of FRβ^+^ myeloid cells in distinct organs of tumor-bearing and healthy animals for inferring the impact of tumorigenesis on RES. The cells were isolated from lungs, liver, spleen, brain, heart, kidneys, lymph nodes, bone marrow, and peritoneal cavity on day 30 after 4T1 inoculation. Infiltration by different types of myeloid cells was drastically enhanced in almost all organs of the tumor-bearing mice compared to healthy controls (Figure 2A). Especially, the granulocyte counts were significantly increased in all organs and tissues investigated. Monocytes reached high levels in lung, liver, spleen, lymph nodes, and peritoneum. Classical CD206^–^ macrophages increased in all organs except bone marrow and peritoneum whereas CD206^+^ macrophages were elevated in lungs, liver, spleen, brain, lymph nodes, and peritoneal cavity (Figure 2A). In the tumor-bearing mice, the organs were highly populated by the granulocytes; therefore, the proportion of granulocytes became significantly augmented compared to that in the healthy animals (Figure 2B). Especially in the lungs, liver, heart, kidneys, and lymph nodes, the percentage distribution of granulocytes was prominent compared to other myeloid cells (Figure 2B).

**Figure 3:**
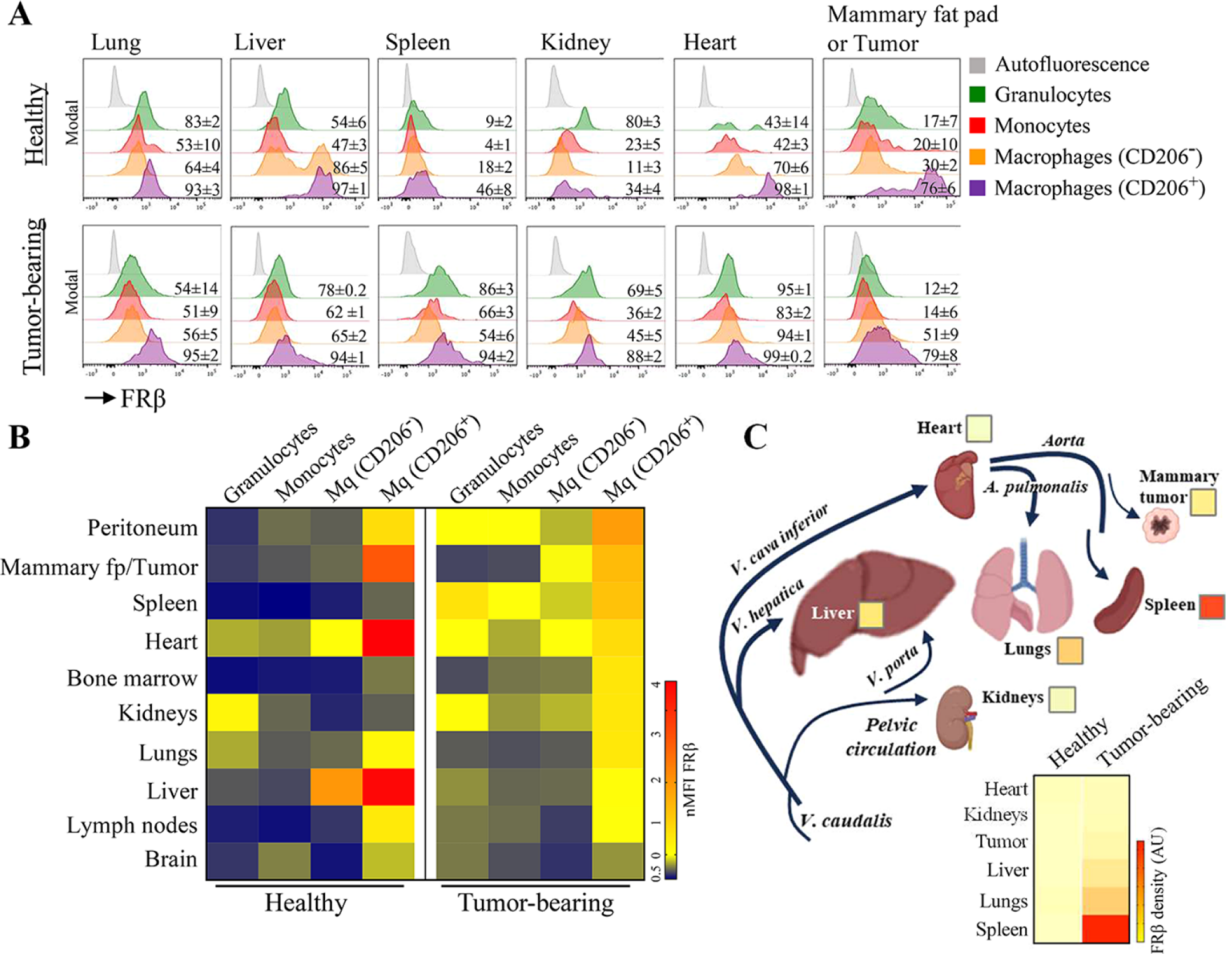
FRβ levels of myeloid cells in healthy and tumor-bearing mice. (A) Representative offset flow cytometry histograms and percentage (average ± SEM) values for the six major tissues studied. (B) Median fluorescence intensity (MFI) heat map for FRβ expression on CD206^+^ macrophages, CD206^–^ macrophages, monocytes, and granulocytes in tissues of healthy control and tumor-bearing (day 30) mice. (C) A schematic showing the major circulation routes through the organs of interest is presented together with a density score calculated for FRβ in each organ. Statistical difference was calculated with Student’s *t* test, (*n* = 6; *p ≤ 0.05, **p ≤ 0.01).

Looking at the distribution of FRβ expression, CD206^+^ macrophages were the myeloid cell group carrying the highest level of the receptor in both healthy and tumor-bearing mice (range, 34–98 and 79–99%, respectively) (Figure 3A and Supporting Figure 3). In healthy mice, almost all CD206^+^ macrophages in the lung, liver, heart, lymph nodes, and bone marrow expressed FRβ (Supporting Figure 3). In terms of the surface expression level, CD206^+^ macrophages localized to liver, heart, and mammary fat pads had significantly higher levels of FRβ (Figure 3B). A significant fraction of CD206-negative macrophages was also FRβ^+^ in the lungs, liver, and heart. Interestingly, a considerable percentage of granulocytes in the lungs and kidneys of healthy individuals expressed FRβ, albeit at low levels (Figure 3B and Supporting Figure 4). While the number of CD206^+^ macrophages was increased in the heart and the liver of tumor-bearing mice (Figure 2A), the surface expression level of FRβ was decreased compared to those in healthy organs (Figure 3B). Additionally, compared to resident CD206^+^ macrophages in healthy mammary tissue, they expressed lower surface levels of FRβ in the tumor tissue (MFI, healthy 6302 ± 3841 and tumor-bearing 3313 ± 1233) (Figure 3B). In the tumor-bearing mice, an overall increase in the percentage of FRβ positivity and in the surface expression levels were observed in all tissues analyzed. Nevertheless, CD206^+^ macrophages carried the highest levels of the receptor. The increase in FRβ expression was remarkable in all cell groups, especially in peritoneum, spleen, and kidneys. Considering both FRβ^+^ myeloid cell percentages and FRβ expression levels, it was concluded that lungs, liver, spleen, kidneys, heart, and breast tumor tissues had the highest amount of FRβ (Figure 3A,B and Supporting Figure 4).

A final reevaluation was performed for determining the FRβ density in each organ by considering the infiltration status of each myeloid cell type per tissue mass, the percentage of FRβ-positive cells, and the surface expression level of FRβ. A score was calculated for a better representation of the FRβ density in each organ. Accordingly, the spleen, lungs, liver, tumor, kidneys, and heart were the organs sustaining the highest FRβ in tumor-bearing mice, respectively (Figure 3C).

All in all, myeloid cell infiltration was significantly increased in all organs and tissues in the 4T1 mammary tumor-bearing mice. Under the influence of tumor, either the percentage of FRβ^+^ myeloid cells or the cell surface expression of FRβ was upregulated on the myeloid cells, especially on the CD206^+^ macrophages residing in distinct organs.

### Association of Organ-Specific FRβ Density and Biodistribution of Folate-Functionalized Cyclodextrin Nanoparticles

**Figure 4:**
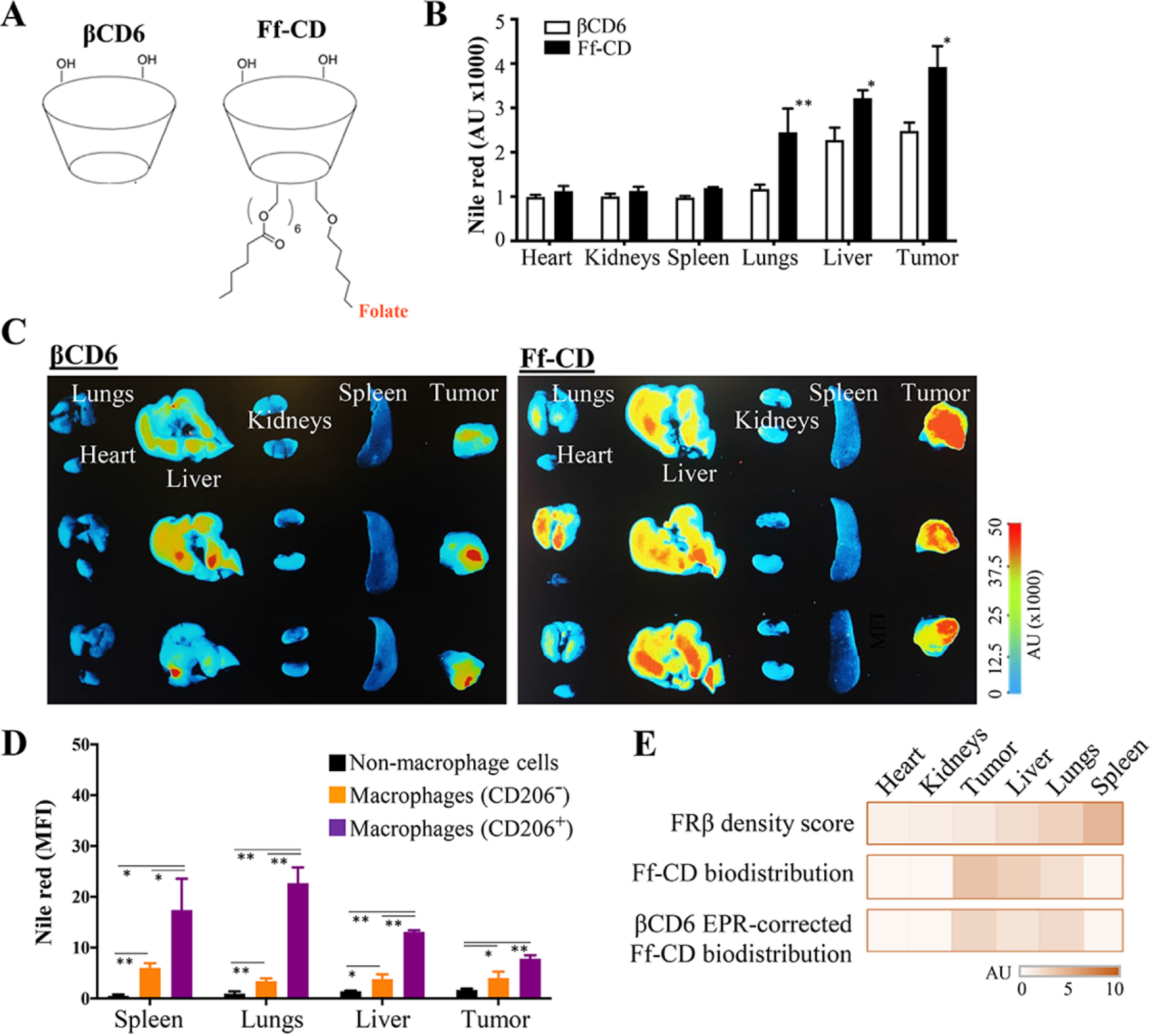
Biodistribution of folate-functionalized nanoparticles in cancer. Folate-functionalized cyclodextrin nanoparticles (Ff-CD) and (6-*O*-methyl)-cyclodextrin (βCD6) nanoparticles, which were used as a prototypic nanoparticle formulation, were loaded with Nile red and injected intravenously via V. caudalis to the tumor-bearing animals. (A) Structure of the nanoparticles is illustrated. (B) Bar graph shows the biodistribution of nanoparticles according to the intensity of Nile red in the major organs studied. (C) Representative organ images showing the biodistribution of Nile red-loaded βCD6 and Ff-CD nanoparticles. (D) The level of Nile red median fluorescence intensity (MFI) in CD206-positive macrophages, CD206-negative macrophages, and in the cells other than macrophages (nonmacrophage cells) is determined by flow cytometry in the cell suspensions prepared from the representative organs. (E) A heat map comparing the density score calculated according to the FRβ expression levels in each organ and the biodistribution of Ff-CD before and after normalization with βCD6 for excluding the influence of EPR. The data are presented as average ± SEM. Statistical difference was calculated with Student’s *t* test, (*n* ≥ 3; *p ≤ 0.05, **p ≤ 0.01).

Next, we sought to investigate the association between the density of FRβ-bearing myeloid cells in the organs and the active-targeting efficacy of the folate-decorated nanoparticles. For this purpose, we preferred to use folate-functionalized cyclodextrin (Ff-CD) and nonfolated control βCD6 nanoparticles which were previously produced and characterized by our group.^35−37^ As a typical nanoparticle, Ff-CD and βCD6 nanoparticles (Figure 4A), whose tumor-targeting capacity have been previously validated,^36^ were loaded with a fluorescent probe (i.e., Nile red) and, herein, used for biodistribution studies. The physicochemical properties of Ff-CD and βCD6 nanoparticles were comparable. The characterization data of CD nanoparticles used after loading with Nile red are outlined in Table 1. In Figure 3C, the calculated FRβ density scores of the organs and the mammary tumor are shown together with the biodistribution routes of the nanoparticles when administered to the systemic circulation via the tail vein. The Ff-CD particles showed significantly (*p* > 0.05) higher uptake in lungs (βCD6, 1170 ± 100; Ff-CD, 2449 ± 535), liver (βCD6, 2276 ± 279; Ff-CD, 3219 ± 179), and tumor (βCD6, 2481 ± 188; Ff-CD, 3924 ± 468) tissues compared to the control βCD6 (Figure 4B,C). At the cellular level in the tested organs, CD206^+^ macrophages internalized Ff-CD particles more efficiently in the spleen, liver, and especially lungs (Figure 4D). The highest Ff-CD signals (MFI, 33 ± 7) were detected from the CD206^+^ macrophages of the lungs. In the tumor tissue, classical macrophages (MFI, 11 ± 3) contained nanoparticles comparable to those of the CD206^+^ macrophages (MFI, 6 ± 1). Ff-CD or βCD6 nanoparticle signals from the nonmacrophage stromal or parenchymal cells were very low (Figure 4D).

The organ biodistribution ranking with Ff-CD nanoparticles presented compatible results with that of the FRβ density score calculated for specific tissues except the spleen and the tumor (Figure 4E). Albeit having the highest FRβ density score, the spleen showed very low nanoparticle interest in biodistribution assays. Consistency between the biodistribution data for Ff-CD and the tumors’ FRβ score was improved when a correction for the EPR effect was performed through subtracting the biodistribution values of βCD6 (Figure 4E). When all of the parameters tested were considered, the most reliable results of folate receptor-targeting were obtained for the lungs (Figure 4E). This finding was further strengthened with the observation that the CD206^+^ macrophages in the lungs of the tumor-bearing mice expressed high levels of FRβ (Figure 3) and very efficiently internalized Ff-CD particles, in vivo (Figure 4D).

Collectively, the folate-functionalized nanoparticles were well-engulfed by CD206^+^ macrophages, which highly expressed FRβ. The FRβ^+^ immune cells found in the tumor microenvironment did not directly influence the tumor-targeting efficacy of the Ff-CD nanoparticles. The lung was determined as a primary target for the folate-functionalized nanoparticles.

## Discussion

**Figure 5:**
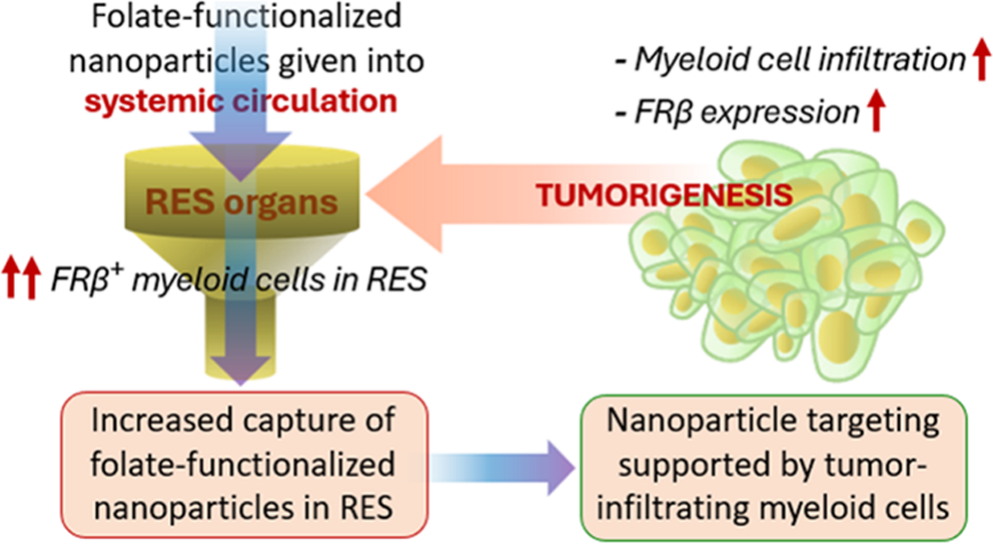
A schematic depicting the major findings of the study. As the tumor progresses, the number of FRβ-expressing myeloid immune cells (especially the CD206^+^ macrophages) is increased in the tumor microenvironment, which creates a favorable target for nanoparticle-mediated drug delivery into the tumor. However, due to the systemic impact of tumor, the organs with RES become more populated by myeloid cells which upregulate FRβ as well; eventually, the folate-functionalized nanoparticles’ efficacy for active targeting of the tumors is hampered.

The reticuloendothelial system (RES) consists of distinct populations of phagocytic cells, primarily monocytes, macrophages, and neutrophil granulocytes located in connective tissue.^33^ These cells significantly contribute to the clearance of macromolecules and solutes in the circulation and tissues; therefore, they play an important role in immune responses against foreign antigens such as bacteria, viruses, and toxins.^32^ The nanoparticles are also recognized as foreign material and removed by RES. Active targeting of nanoparticles may be a solution for bypassing the organs rich in RES and increasing the efficacy of drug delivery to tumors.^33^ Decoration with folate is a promising functionalization for the nanoparticles since FRs are upregulated to meet the increased metabolic demand for folic acid in cancer.^30^ Under physiological conditions, there were very limited numbers of tissue-resident CD206^+^ subset of macrophages that were positive for FRβ. In our study, the myeloid cells were increased gradually in the breast microenvironment during the tumor formation and almost all organs became significantly infiltrated by granulocytes, monocytes, and macrophages systemically in progressed disease. Moreover, FRβ expression was upregulated on the tumor-infiltrating myeloid cells. This was regarded as a positive phenomenon which would enhance the targeting efficacy of folate-functionalized nanoparticles. On the other hand, the myeloid cells and their FRβ expression were also increased in various RES organs of the tumor-bearing mice. Due to the tumor burden, the negative impact of RES became more profound through capturing the nanoparticles and preventing them from reaching the target. In cancer patients, various tissues (other than the organs containing primary tumors or metastasis) become populated with high numbers of myeloid cells due to increased hematopoietic activity.^41^ The major findings of this study were summarized in Figure 5. According to our results, increased expression of FR in RES was associated with enhanced retention of folate-functionalized nanoparticles in the lungs, liver, and tumor. Here, we discuss the interrelationship between biodistribution of FRβ^+^ myeloid immune cells and the biological parameters modulating the targeting efficacy of the folate-functionalized nanoparticles.

Not only the malignant cells but also the immune cells become metabolically altered in cancer.^8^ Myeloid cells and tissue-resident macrophages form the first line of defense in response to microbial insult or tissue damage.^42^ Metabolic activity and the need for folic acid derivatives are increased in the myeloid cells due to activation and/or infiltration of tumors. Therefore, expression of high affinity folate receptors is upregulated.^43^ Therefore, not only the tumor cells but also the myeloid cells are favorably targeted by the folate-functionalized delivery systems.

The route of systemic administration, which is typically intravenous injection via caudal vein in mice models, leads to an initial deposition of nanoparticles in the liver where the capillary circulation paths facilitate the capture of (macro)molecules by resident phagocytes.^33^ In the breast cancer model used, the liver possessed increased numbers of myeloid cells with elevated FRβ. Expectedly, the retention of Ff-functionalized nanoparticles was increased in the liver of tumor-bearing animals. Then, the nanoparticles who reach the heart are ejected into the circulation albeit a major blood flow is directed into the lungs, which contain a fine network of microvessels.^33^ Augmented numbers of FRβ^+^ myeloid cells were detected in the lungs of tumor-bearing animals; accordingly, this organ specifically became a target for the cyclodextrin nanoparticles conjugated with folate. Both the liver and especially the lungs are the primary organs for metastasis, in 4T1 breast cancer.^39,40,44^ In the progressed disease (day 30), the metastatic foci in these organs may have altered the vasculature and created an environment that favors the EPR effect. Intriguingly, when the biodistribution of Ff-CD nanoparticles was corrected by using the data from nontargeted nanoparticles (βCD6), in contrast to the tumor and the liver, active targeting of Ff-CD was more successful in the lungs although the nanoparticles were specifically engulfed by the FRβ^+^CD206^+^ macrophages. Therefore, the folate-conjugated nanoparticles had a greater interest in lung tissue potentially due to increased targetability of FRβ expressed by myeloid cells. This was in accordance with our previous studies where folate-conjugated CD nanoparticles proved to be efficient for hindering breast cancer metastasis to the lungs.^36,37^

The remaining nanoparticles that are not captured by RES in the liver and the lungs reached the breast tumor. Normal breast tissue harbored FRβ-expressing macrophages; nevertheless, these cells were highly populated in the progressing tumor. Therefore, the increased FR levels together with the leaky vasculature of the tumor site established a favorable target for Ff-CD nanoparticles. Intriguingly, the signals from the nanoparticles, which reached the tumor, were not restricted to the myeloid cells. Even though not tested in our study, other FRs or folate transporters which can be upregulated upon tumorigenesis might have enhanced the nanoparticle uptake by other cells of the tumor microenvironment.^30^ For example, metabolomic analysis of breast cancer cells upon exposure to the folate-conjugated CD nanoparticles indicated early apoptosis and modulation of hexose metabolism.^45^

As an immune organ, the spleen was highly populated by the myeloid cells in response to tumorigenesis.^41^ Enlargement or histological alterations of the spleen due to extensive accumulation of myeloid cells has been previously reported in mouse cancer models and in cancer patients.^39,46^ In the spleen, blood flows through a maze of sinusoids lined by endothelial cells and the gaps in the endothelium create a filter-like structure that enables clearance of large bodies such as damaged erythrocytes and cell debris by phagocytes.^41^ In our study, the spleen displayed the highest FRβ density score in tumor-bearing animals. Expectedly, a high FRβ density score was calculated for the spleen since it is an immune organ and an excess number of myeloid cells constitute the spleen tissue. Nevertheless, Ff-CD nanoparticles were not highly accumulated into the spleen; however, a considerable amount of nanoparticles were trapped in FRβ^+^CD206^+^ macrophages. Cyclodextrin nanoparticles used in this study had a size range between 100 and 200 nm which can reduce splenic retention of nanoparticles.^36,37^ Moreover, it can be speculated that the sinusoidal structure in the enlarged spleen may limit the access of nanoparticles to RES cells in cancer. Previous research has demonstrated that nanoparticles measuring 100–200 nm or smaller exhibit enhanced permeability through the endothelial fenestrae, constituting the splenic sinuses’ filtering interface. In contrast, larger particles undergo gradual clearance by red pulp macrophages.^47^ Moreover, owing to the highly negatively charged nature of the vascular endothelial luminal surface and the membranes of spleen cells, particles with negative charge experience hindered binding affinity.^32^ Even though the FRβ score was high, its anatomical structure of the spleen may have limited the capture of functionalized CD nanoparticles when compared to the other organs, where the RES is tightly packed to filter smaller molecules.

Upregulation of FRβ on myeloid immune cells in cancer can serve as an advantage for targeting immunotherapy drugs into the tumor microenvironment.^12^ Reprogramming the immunosuppressive cells for gaining antitumor capacities is a promising therapy approach in cancer. Folate-mediated immune intervention treatments for cancer have been reported previously.^12,31,48−50^ From an alternative point of view, our data support that the myeloid cells infiltrating many organs, such as the liver and lungs, which are primary targets for cancer metastasis, and the CD206^+^ tumor-associated macrophages (TAMs) can be more efficiently targeted with folate-functionalized nanoparticles for immunotherapy approaches. It should be noted that in healthy animals, the amount of FRβ^+^ myeloid cells in various organs is limited, and the systemic biodistribution of folate-functionalized nanoparticles might not reflect in situ condition in cancer. Moreover, myeloid cells are implicated in the pathogenesis of many inflammatory diseases; therefore, folate-functionalized nanoparticles loaded with immunomodulatory drugs might be used for targeting the FRβ^+^ myeloid cells in many organs and may possess further relevance for clinical applications.

## Conclusions

Notwithstanding many in vitro reports that support the efficacy of folate-targeting, in vivo studies are essential to better define the limitations of this active drug delivery approach. Folate receptors are upregulated to meet the increased need for folate due to continuous cell proliferation and reprogramming of metabolic activity during tumor formation. In a cancer model, our study reported the distribution and amount of FRβ-expressing myeloid cells in distinct organs and in mammary tissue during tumorigenesis. Targeting folate-related pathways or receptors is a promising therapeutic approach for cancer. Albeit being a promising drug delivery and tumor-targeting strategy, the nanoparticles decorated with folate have certain limitations such as increased clearance by RES myeloid immune cells that upregulate FRβ in cancer.

## Supporting Information Available

Additional information on the gating strategy used for immunophenotyping analyses, and additional results on the animal weight and the tumor size, the expression of FR isoform genes, additional data on the FRβ expression in myeloid cell subpopulations in distinct tissues and representative offset flow cytometry histograms (PDF)

## Abbreviations

FR: folate receptor
FRα: folate receptor alpha (α)
FRβ: folate receptor β (β)
FRγ: folate receptor gama (γ)
FRδ: folate receptor delta (δ)
RES: reticuloendothelial system
RNA: ribonucleic acid
DNA: deoxyribonucleic acid
Treg: regulatory T cell
TAM: tumor-associated macrophages
CD: cyclodextrin
βCD6: 6-*O*-methyl β-CD
Ff-CD: folate-functionalized cyclodextrin
SEM: standard error mean
MFI: median fluorescence intensity
