# Supplementary material for: Folate Receptor β (FRβ) Expression on Myeloid Cells and the Impact of Reticuloendothelial System on Folate-Functionalized Nanoparticles’ Biodistribution in Cancer
Source: Mol Pharm. 2024 Aug 6;21(9):4688–99. doi: 10.1021/acs.molpharmaceut.4c00663 (PMC11372836; doi:10.1021/acs.molpharmaceut.4c00663)
Supplement: Supplementary file 1 — mp4c00663_si_001.pdf [file mp4c00663_si_001.pdf]

Folate Receptor β (FRβ) Expression on Myeloid Cells and the Impact of Reticuloendothelial System on Folate-functionalized Nanoparticles' Biodistribution in Cancer

Sibel Goksen[1], Gamze Varan[2], Erem Bilensoy[3], Gunes Esendagli[1,4,5*]

[1]Department of Medical and Surgical Research, Institute of Health Sciences, Hacettepe University, Ankara, 06100, Türkiye.

[2]Department of Vaccine Technology, Hacettepe University Vaccine Institute, Ankara, 06100, Türkiye.

[3]Department of Pharmaceutical Technology, Faculty of Pharmacy, Hacettepe University, Ankara, 06100, Türkiye.

[4]Department of Vaccinology, Hacettepe University Vaccine Institute, Ankara, 06100, Türkiye.

[5]Department of Basic Oncology, Hacettepe University Cancer Institute, Ankara, 06100, Türkiye.

Corresponding author: Gunes Esendagli, PhD.

Hacettepe University Cancer Institute, Department of Basic Oncology

06100, Sihhiye, Ankara - Türkiye

Fax: +90 312 324 20 09

*E-mail: gunese@hacettepe.edu.tr

Supplementary Figures

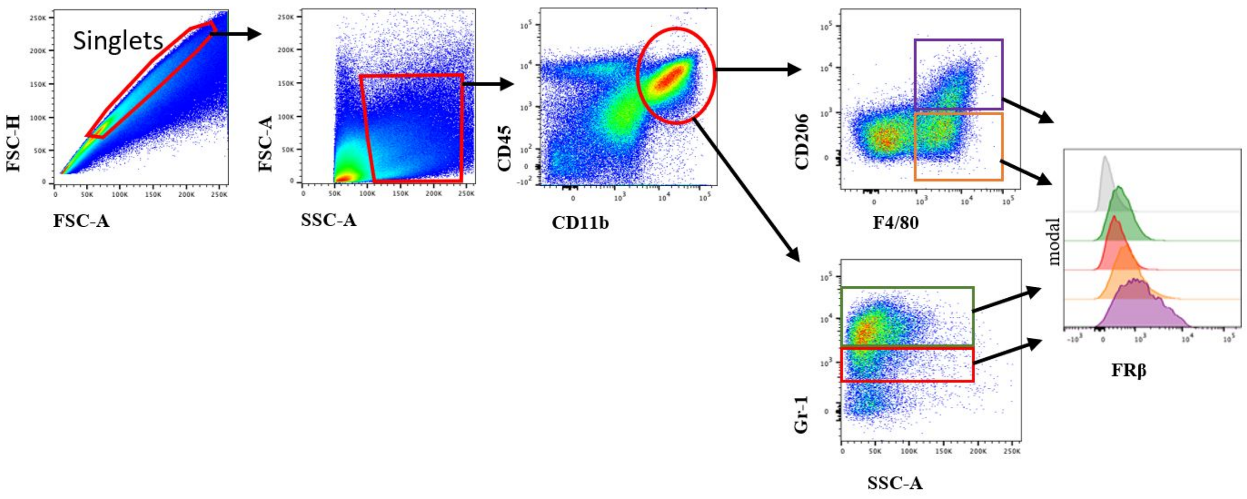

Supplementary Fig. 1. Gating strategy used for the immunophenotyping analyses by flow cytometry. The dot-plots show the cell suspensions prepared from the tissues which were gated as single events (doublet discrimination) and after selecting populations with appropriate cell size and granularity, $CD45^{+}CD11b^{hi}$ myeloid immune cells were gated. Then, macrophages were detected as $F4/80^{+}CD206^{-}$ and $F4/80^{+}CD206^{+}$ populations. FRβ expression was analyzed and compared in each population and presented as an offset image.

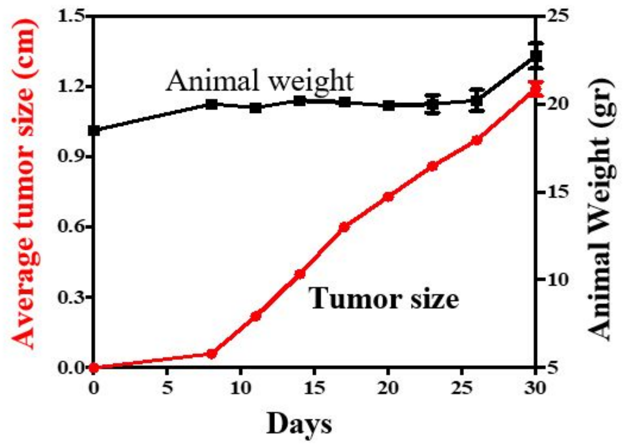

Supplementary Fig. 2. The animal weight and the breast tumor size followed up for thirty-day-long period after the inoculation of 4T1 breast cancer cells are plotted.

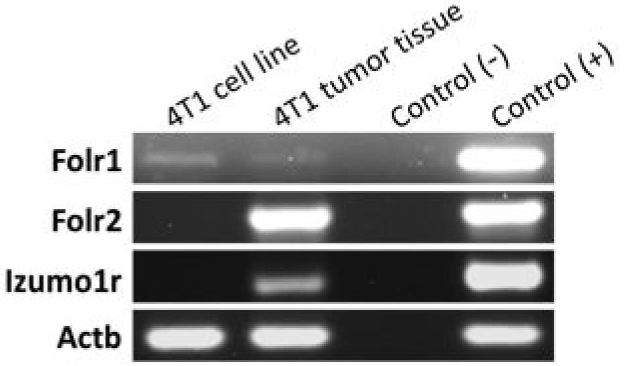

Supplementary Fig. 3. The expression of Folr1, Folr2 and Izumor1r genes encoding FRα, FRβ and FRδ, respectively, was studied by RT-PCR. Total RNA was extracted from 4T1 cell line cultured in vitro and from the tumor tissues established with 4T1. Please note that the tumor tissue constitutes not only the tumor cells ut also the elements of the microenvironment such as stromal cells and immune cells. B-actin gene (Actb) was used as a housekeeping control. The gene amplification products were resolved on agarose gel electrophoresis and the bands were documented.

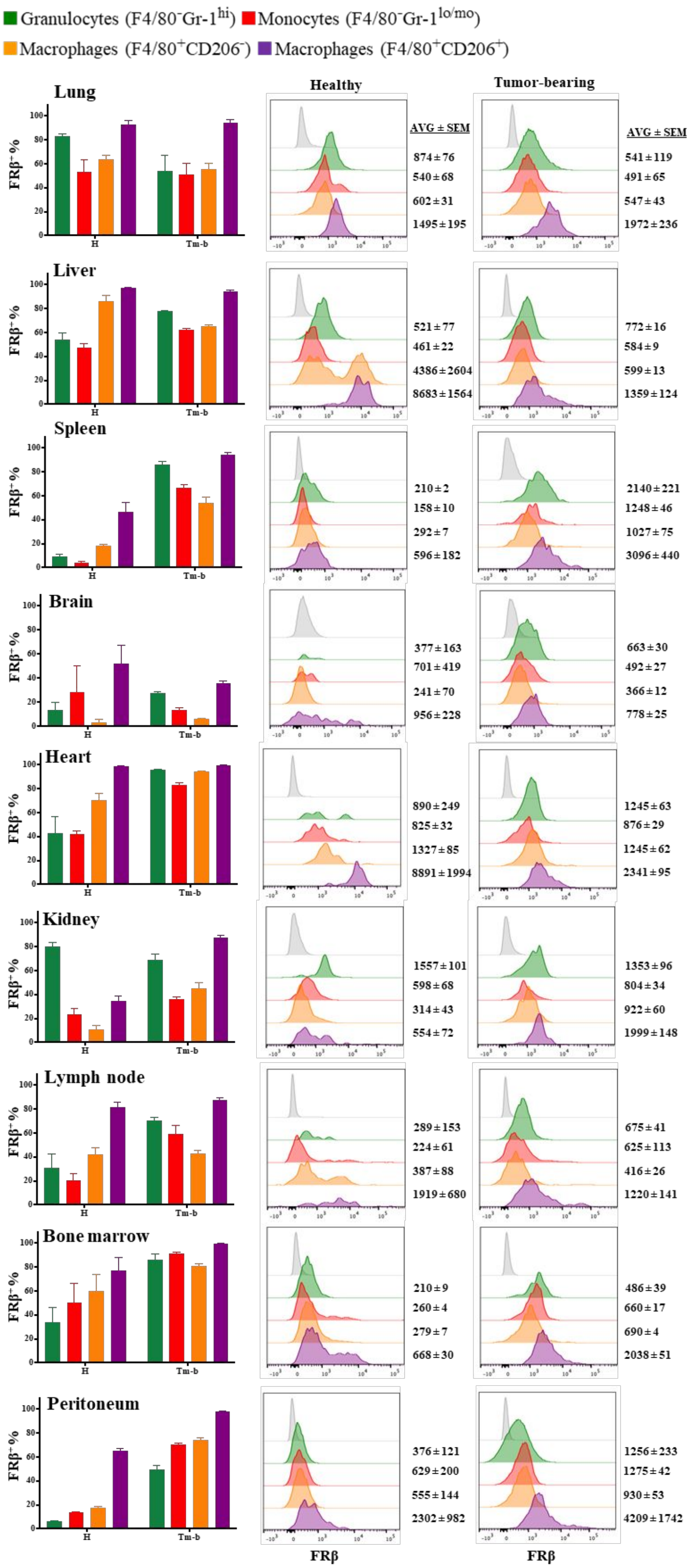

Supplementary Fig. 4. A) Bar graphs show the percentage of granulocytes, monocytes, CD206⁻ macrophages, and CD206⁺ macrophages expressing FRβ in distinct tissues of healthy and tumor-bearing animals on day 30. **B)** Representative offset flow cytometry histograms and MFI (average ± SEM) values for the tissues studied. The data are presented as average ± SEM.
